# A life‐time of hematopoietic cell function: ascent, stability, and decline

**DOI:** 10.1002/1873-3468.14843

**Published:** 2024-03-05

**Authors:** Anna Popravko, Lorna Mackintosh, Elaine Dzierzak

**Affiliations:** ^1^ Institute for Regeneration and Repair University of Edinburgh UK

**Keywords:** aging, development, fate, hematopoiesis, hemogenic endothelial cell

## Abstract

Aging is a set of complex processes that occur temporally and continuously. It is generally a unidirectional progression of cellular and molecular changes occurring during the life stages of cells, tissues and ultimately the whole organism. In vertebrate organisms, this begins at conception from the first steps in blastocyst formation, gastrulation, germ layer differentiation, and organogenesis to a continuum of embryonic, fetal, adolescent, adult, and geriatric stages. Tales of the “fountain of youth” and songs of being “forever young” are dominant ideas informing us that growing old is something science should strive to counteract. Here, we discuss the normal life stages of the blood system, particularly the historical recognition of its importance in the early growth stages of vertebrates, and what this means with respect to progressive gain and loss of hematopoietic function in the adult.

## Graphical abstract

Here we review the aging of the hematopoietic system from its earliest stages in the embryo through fetal and adult life. We focus on the waves of hematopoietic cell generation during embryonic life and how cells of varying lineages, functions and life spans contribute to the normal development of the adult blood system and its progressive gain/loss of function in the aged adult.

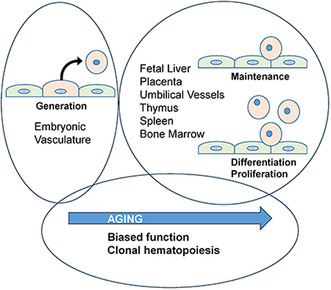

## Abbreviations

**CH**, clonal hematopoiesis

**E**, embryonic day

**EMP**, erythroid‐myeloid progenitors

**HEC**, hemogenic endothelial cell

**HPC**, hematopoietic progenitor cells

**HSC**, hematopoietic stem cell

**LMPP**, lymphoid multipotent progenitors

**MPP**, multipotent progenitors

## Generation of blood, circulation, and wide diversity of hematopoietic cells

Developmental origins of red and white blood cells, and the potent hematopoietic stem cells (HSCs) that continuously replenish the adult blood system, have intrigued research scientists and clinicians for centuries. As early as the 3rd century BC, blood was conceptualized by Aristotle and the ancient Greeks as essential to life, with the heart and contiguous vessels distributing it to nourish the organs of the body. Indeed, the term for red blood cell production is derived from Greek “erythro” meaning red and “poiesis” to make. Proof for the circulation of red blood cells throughout the body was realized in the 17th century, with the publication of William Harvey's famous work, “De Motu Cordis et Sanguinis”—on the motions of the heart and blood in animals. To distinguish hypotheses from facts, Harvey introduced scientific methods into biology and science [1, 2]. He analyzed the structure and contractile action of the heart and the consequential pulsatile circuiting of blood throughout the body. His estimations of blood quantity in the heart, veins, and arteries, challenged the prevalent view of the time that the liver was the origin of venous blood. Prospective experimentation involving the tying of ligatures around the vessels of fish and in the human arm further implicated the circulation of blood through the body. This was followed by the discovery of valves by Fabricius [3] and the discovery of capillaries connecting arteries into the veins by Malpighi [4]. Harvey only accepted the results of his research when they were also confirmed in control experiments, giving rise to evidence‐based medicine. Interestingly, he also made significant contributions to embryology and adopted the notion of successive stages of dynamic development, although hematopoietic cell development was generally unstudied until much later.

Fast forward to the 20th century, when innovative cellular and molecular experimental strategies expanded our understanding of blood and the hematopoietic system. The white blood cell lineages (phagocytic cells, innate and adaptive immune cells) were originally distinguished microscopically by their lack of red color and unique morphologic characteristics, with microscopists studying sectioned vertebrate embryos in the late 19th and early 20th centuries identifying clusters of spherical hematopoietic‐like cells juxtaposed to the major vasculature [5]. Later, *in vitro* cultures of blood and bone marrow cells, *in vivo* transplantations, clonal/molecular marking, and lineage tracing began to unravel the hematopoietic differentiation hierarchy and the rare HSCs that are at the foundation of the adult hematopoietic hierarchy. More recent studies have highlighted the fact that the hematopoietic system undergoes multiple developmental changes with periods of stability in midlife followed by decline during aging [6, 7].

## Hematopoietic birth is multilayered and occurs in waves of blood cell generation

Complex tissue formation occurs in coordinated waves of cell generation during embryonic and fetal life to culminate in an intact functioning adult organism. The first waves of specific (tissue/cell) formation consist of cell types that support the simple, and often temporary, cellular needs of the growing embryo. Co‐ordinately established microenvironments are conducive to the generation and expansion of more complex cell types functioning at fetal stages, with a final wave culminating in the generation of long‐lived self‐renewing tissue‐specific multipotent progenitors or stem cells to sustain the physiology and function of whole tissues throughout adult life.

**Fig. 1:**
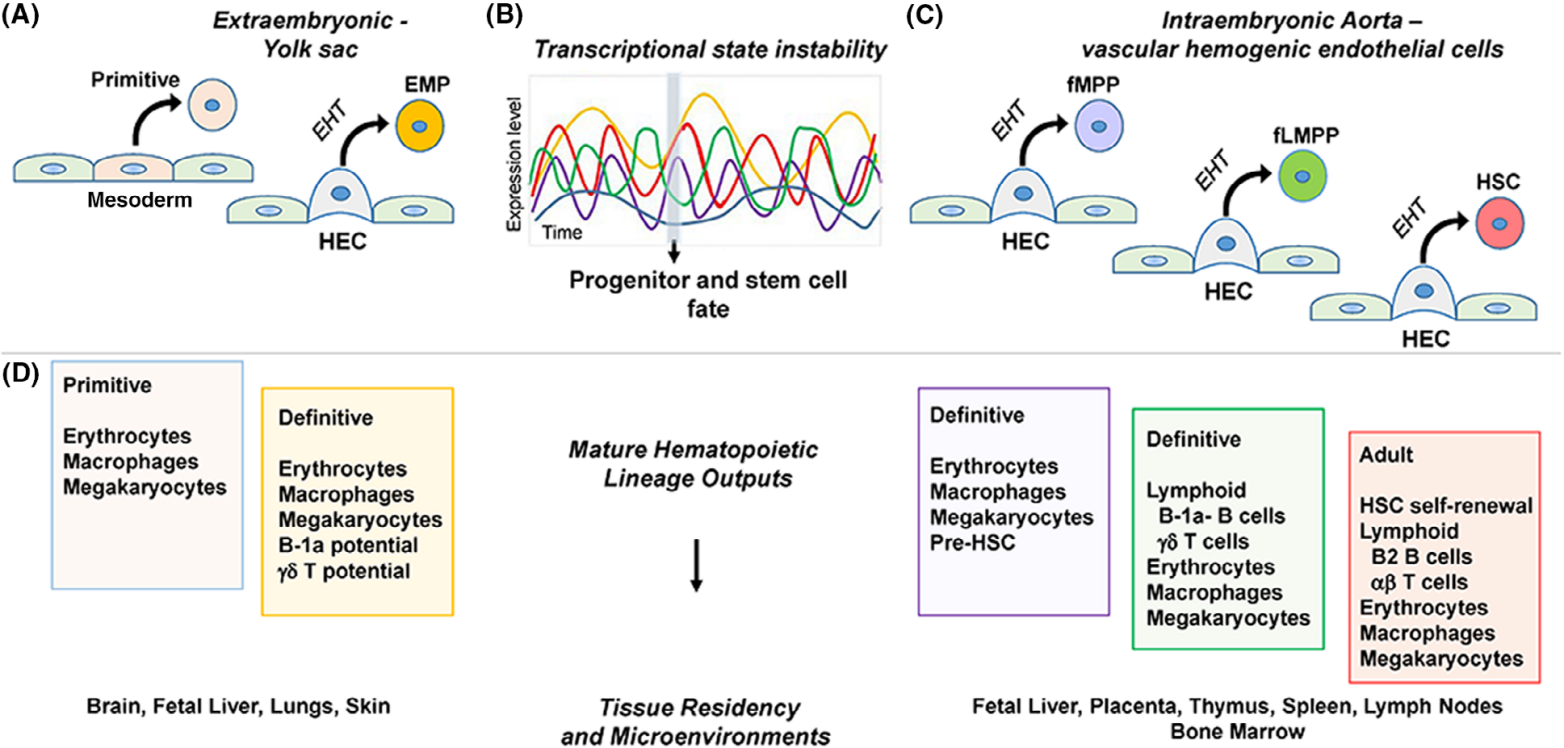
Diversity of the hematopoietic cells produced during the multilayered generation of the blood system. (A) Primitive erythrocytes, macrophages, and megakaryocytes are directly generated from yolk sac mesoderm. Erythroid‐myeloid progenitors (EMP) are bi‐potential hematopoietic progenitors that arise from hemogenic endothelial cells (HEC) of the yolk sac. (B) A combination of pivotal transcription factors and regulators intrinsically control the generation and fate of hematopoietic progenitors and stem cells as they emerge from HECs. Each individual cell is characterized by instability and asynchrony of the group of pivotal factors (level changes over time) that stochastically establish its fate. (C) Intraembryonic aortic vascular hemogenic endothelial cells (HEC) generate cells fated to be embryonic/fetal multipotent progenitors (fMPP), embryonic/fetal lymphoid multipotent progenitors (LMPP) and definitive hematopoietic stem cells (HSCs). (D) The mature hematopoietic cells produced from the yolk sac and aortic generated precursors and their tissue residency through development. Differentiation and maintenance of the cells is within a variety of microenvironmental niches of the fetus and adult.

A good example of this is seen within the kidney, which undergoes three developmental stages: pro‐, meso‐, and meta‐nephric stages. These take place in virtually the same localization within the embryo but originate at different times from distinct cells [8]. The complexity in molecular programs and gene regulatory networks increases as the organism grows to incorporate, designate, and support more advanced physiologic tissue functions. Similarly, different developmental origins of muscle stem cells are likely the source of functional myogenic heterogeneity in the adult [9]. While the embryonic development of the adult hematopoietic system was once thought to originate singularly from fully potent HSCs generated in the yolk sac, current research describes the generation of a wide spectrum of the hematopoietic cells of varying potentials and functions prior to definitive HSC generation [6]. HSC‐independent blood generation constitutes the first two waves of hematopoietic cell birth (embryonic and fetal) [10, 11, 12], with the adult wave of HSC generation as the third and most long‐lived (Fig. 1).

Non‐mammalian vertebrate embryo studies highlighted the differential potential of embryonic tissues and vasculature for waves of hematopoietic cell generation. Avian and amphibian grafting and marking experiments [13, 14] revealed the distinct generation of the short‐lived embryonic red cells from the yolk sac and the generation of the long‐lived adult hematopoietic system from the dorsal aorta during early/mid‐gestation [15, 16]. In the mouse, the long‐lived HSCs of the adult hematopoietic system (as defined by *in vivo* long‐term multilineage engraftment of adult recipients) were discovered to be generated in the embryonic aorta [17, 18, 19] and vitelline/umbilical arteries [20], subsequent to the generation of embryonic erythrocytes, macrophages and megakaryocytes in the yolk sac. Genetic marking experiments confirmed the independent origins and waves of hematopoietic cell generation in the mouse [21, 22, 23]. In humans, the embryonic aorta has also been shown to give rise to the first multilineage hematopoietic repopulating cells [24, 25, 26, 27]. Therefore, the developmental ascent of the hematopoietic system consists of layers of successively (and independently) generated cell types with distinctive and increasingly complex hematopoietic potentials and functions.

## Molecular and biophysical characteristics of blood associated with different life stages

### Mature lineages in embryonic and fetal/adult stages

During development, the rapid generation of myeloid, megakaryocytic, and lymphoid cells in an HSC‐independent manner is thought to be a short‐cut mechanism by which vascular endothelial/hemangioblastic precursors directly differentiate to mature blood cells or produce short‐lived intermediate cell types (Fig. 1A,C; embryonic/fetal hematopoietic progenitor cells (HPC)). Yolk sac and primitive streak mesodermal cells (Flk+ and brachyury+) in the mouse possess potential for endothelial/hematopoietic transition as early as embryonic day (E) 7.5 [12]. Depending upon temporal/spatial generation [2], the earliest mature blood cells differ from those in the adult in cellular and molecular characteristics, function, and persistence (Fig. 1D).

The best and most studied example is the erythroid lineage. At embryonic day (E) 7.5 in the mouse and day ~16–20 in the human conceptus, erythrocytes with an embryonic/primitive globin gene program are produced directly, rather than through differentiation of intermediate hematopoietic progenitors [28]. These are the only erythrocytes that retain their nucleus and proliferative capacity when they enter the circulation [29]. At E8.5 in the mouse, prior to HSC generation, erythroid‐myeloid progenitors (EMP) are generated in the yolk sac [10]. During subsequent colonization of these EMPs in the fetal liver, they differentiate toward the definitive erythroid lineage [30], which supports the metabolic needs of the growing fetus [31]. In contrast, HSCs, which also colonize the fetal liver, do not produce mature blood cells at this stage [32].

At birth, definitive erythropoiesis shifts towards the bone marrow and spleen, where homeostatic controls maintain the steady production of HSC‐derived mature erythrocytes (1% replacement/day). This complex process involves around 10 cellular intermediates from the original HSC to the final mature erythrocyte. Erythrocyte differentiation occurs within erythroblastic islands, specialized microenvironmental niches supported by a central macrophage [28]. The molecular changes accompanying this extended differentiation process (occurring over ~1 week in human) include genes controlling cell division, survival, size reduction, shape change, metabolism, and enucleation. Of prime importance to adult is the oxygen‐carrying capacity of circulating erythrocytes established by the developmental molecular switch within the globin gene loci to the adult globin genes, allowing a transition to HbA production within the first 6 months of life [33].

Other primitive cell types made in mouse and human conceptuses include macrophages, megakaryocytes, and mast cells. While primitive megakaryocytes can make platelets, their precise role and the role of mast cells in fetal stages remains undetermined [34, 35, 36]. In contrast, primitive macrophages are important in embryonic tissue remodeling and lymphatic development during fetal life. The yolk sac‐generated macrophages display remarkable longevity and persist into adulthood in various tissues, including within the brain as microglia [37]. They are produced independently of a monocyte precursor, which is characteristic of EMP‐ and HSC‐generated macrophages [6]. HSC‐generated monocyte‐derived macrophages provide a range of functions related to innate immunity in adult life, whereas the yolk sac‐derived tissue‐resident macrophage populations demonstrate marked diversity in both phenotype and function within their respective organs systems [38]. The processes driving this heterogeneity both between tissue‐resident populations and with their monocyte‐derived macrophage counterparts remain unclear, as does the importance of their distinct developmental origins in terms of mature cell functions.

Similarly, the lymphoid lineages arise from different embryonic tissues prior to HSC development [39], such as the B‐1a B lymphocyte subset [40]. B cell potential is detected as early as E8.5 intraembryonically and are detected in the fetal liver [41]. Immune‐restricted lymphoid‐primed progenitors are found in the extra‐embryonic yolk sac but the progenitors do not persist into adulthood [42]. Also, lineage tracing has identified an atypical fetal liver HSC that contributes to the B‐1a cell population [43]. In contrast, the more mature B2 B cell subset differentiates from definitive HSCs.

Prior to HSC development, T lymphocyte potential is detected in the E9.5 yolk sac and, under strong Notch1 signaling, in the E9.5 aorta [39, 44, 45]. Early embryonic/fetal T cells characteristically express gamma/delta T cell receptors, whereas T cells originating from HSCs express alpha/beta receptors. Interestingly, a Rag1+ progenitor with myeloid and T cell potential was found to colonize the thymic rudiment at E11.25 [46]. Hence, hematopoietic cells of myeloid and lymphoid lineages are not only generated by HSCs, but are generated prior to HSC development from embryonic and fetal progenitors of the yolk sac, intraembryonic vasculature, and liver.

## Cell intrinsic and extrinsic aspects of development—achieving stability

Intrinsic molecular programs are at the foundation of cellular identity and functional properties. To understand the basis of primitive and definitive hematopoietic cell generation, proliferation, differentiation, and function, it would be ideal to identify the specific transcriptomes and proteomes of all primitive and definitive hematopoietic cells, intermediates, and mature cell outputs. Based on cell frequencies in populations isolated by surface proteins expression, some transcriptional nodes have been identified within a hematopoietic differentiation continuum [47]. However, single cell technologies have revealed heterogeneity within highly enriched phenotypic hematopoietic progenitors and HSCs, suggesting that multipotent progenitors and stem cell are in semi‐stable transcriptional states (Fig. 1B) [48, 49, 50]. Small or hemizygous changes in transcription factor expression levels significantly affect HSC quality, quantity, and fate [51, 52]. Also, various states of cell cycle are implicated in dormancy, quiescence, or proliferation [53, 54]. What is it that determines the intrinsic program of a primitive blood cell or definitive hematopoietic cell progenitor/intermediate/stem cell, whether it persists or is extinguished and differentiates to perform a specific function at distinct life stages?

Supportive properties extrinsic to hematopoietic cells (mature, progenitor, stem) are found in the special microenvironments and niches that change and are replaced during successive life stages. The yolk sac microenvironment, mainly consisting of mesoderm, endoderm and endothelial cells is the most active at the earliest periods of development, but goes extinct in late mammalian gestation when EMP production terminated [10]. While yolk sac‐generated primitive hematopoietic cells are dispersed through the fetal circulation, EMPs emigrate to the fetal liver microenvironment where they differentiate to meet the organismal blood demands of the growing fetus. Around this time point, the intraembryonic splanchnopleural mesodermal microenvironment establishes hemogenic endothelial cells (HEC; Fig. 1C) that give rise to more complex definitive multipotent hematopoietic progenitors and HSCs [18, 40, 55]. These hematopoietic cluster cells do not differentiate within the aortic vascular niche, but rapidly transit through the circulation to colonize the fetal liver where they will expand [56]. Additional colonization of the placenta has been suggested, with placental progenitors and HSCs significantly increasing in number over gestational time [57, 58, 59]. Unlike EMPs however, the multipotent progenitors (MPP) and HSCs do not significantly contribute to the fetal blood system [22]. Around the time of birth, the fetal liver microenvironment undergoes a transition from a highly hematopoietic differentiative tissue for EMPs and a supportive microenvironment for HSC/progenitor expansion, to a non‐hematopoietic microenvironment that becomes filled with hepatocytes, stellate and globlet cells. Fetal liver HSCs and HPCs emigrate to the bone marrow microenvironment through the circulation, and placental HPC and HSC are released into the umbilical vessels (Fig. 1D).

Considered as the secondary hematopoietic tissues established during fetal stages (Fig. 1D), the thymus evolves through several hematopoietic cell seeding events in which hematopoietic cells [60], in parallel with the thymic stromal microenvironment, engage in crosstalk to influence each other and establish the supportive microenvironment for the differentiation of T cell precursors to mature CD4 and CD8 T lymphocytes. Later in adolescent life, the thymus involutes and declines in activity. Similarly, the lymph nodes and spleen also develop through emigration and crosstalk events between niche and hemato‐lymphoid cells [61], but do not involute and are active through adult life.

Around the time of birth, the specialized microenvironment of the adult bone marrow has formed and takes over the support and life‐long maintenance of HSC and progenitors and the daily production of mature blood cells. It is a robust structural environment that provides necessary cytokine and endocrine support for HSCs and consists of two niches, the endosteal and the central vascular niche [62]. The osteoblasts and endothelial cells of the endosteal niches promote HSC quiescence and are involved in regenerative hematopoiesis and lymphopoiesis, while the central vascular (sinusoid/arteriole) niches are involved in the daily production and transmigration of blood cells and myelopoiesis. Additional cells within the bone marrow also play a role in the wider control of blood cell production, such as the sympathetic nervous system (diurnal cycle and trafficking) [63, 64], stromal cells (mesenchymal, adipocytes, pericytes) [65] and hematopoietic cells themselves (inflammation and stress) [66].

In general, hematopoiesis and the hematopoietic microenvironments enter a stage of relative stability and homeostasis during reproductive years of life. Only upon extrinsic changes such as stress, infection, genetic disease, pregnancy, nutritional deficits, and other emergencies does the hematopoietic system respond and adapt to the provide for the organismal needs of the individual.

## HSC aging: loss of function, limited lifespan, and competition

With advanced single cell technologies at both the functional and omic level, fundamental differences in the blood system of aged adults, as compared to young adults, are becoming more apparent. Two main features of aging and the decline in robust hematopoiesis have emerged and are based within the cohort of bone marrow HSCs in the adult. The first involves changes in the lineage differentiation bias of aged HSC cohorts and the second involves competitive advantage and clonal hematopoiesis that advances with aging.

**Fig. 2:**
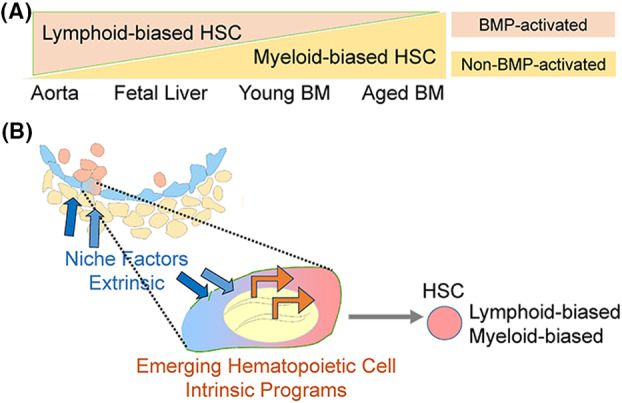
Distinct hematopoietic stem cells (HSCs) give hematopoietic lineage outputs that are balanced or biased. (A) Stage‐specific lineage‐biased mature hematopoietic cell outputs of HSC shown as proportions of lymphoid‐biased (orange) and myeloid‐biased (beige) HSCs. Proportions of HSCs found in different hematopoietic tissues (embryonic aorta, fetal liver and bone marrow (BM) of young and aged adults) show a shift from predominance of lymphoid‐biased to myeloid‐biased HSCs over developmental time and tissue residence. Activation by developmental signaling molecule BMP4 discriminates two HSC types (BMP‐activated and non‐BMP‐activated). (B) Extrinsic developmental microenvironment/niche factors (blue arrows) signal hemogenic endothelial cells in the process of becoming HSCs to influence intrinsic transcriptional (and epigenetic) programs that contribute to HSC‐bias toward lymphoid lineage and/or myeloid lineage output.

Heterogeneity in functional bone marrow HSCs and their lineage differentiation potential as determined in clonal mouse bone marrow HSC repopulation studies was first observed by Muller‐Sieburg and rigorously confirmed by others [67]. The differentiation potential of clonal HSCs (as measured by lymphoid and myeloid lineage representation in peripheral blood) was found to be balanced, lymphoid‐biased or myeloid‐biased. Interestingly, most HSCs found in aged individual are myeloid‐biased, whereas in young and middle‐aged individuals they are mainly lymphoid‐biased or balanced in output (Fig. 2). Transcriptomic studies have attempted to identify the intrinsic molecular differences in these HSCs. Profiling, together with genetic and functional tests indicate the involvement of cytokine/growth factors and receptors such as BMP (Fig. 2A), TGFbeta1, and PDGFRbeta [68, 69, 70], suggesting HSC heterogeneity is based on their endothelial cell origins and/or their journey through to the various developmental microenvironments (Fig. 2B). Indeed, lineage tracing studies demonstrate that lineage‐bias shift in the cohort of HSC found during aging could be in some cases attributed to the intraembryonically derived HSC‐independent MPPs/LMPP that can provide both lymphoid and myeloid output to middle‐age [22, 71].

Although adult HSCs occupy the bone marrow throughout adulthood and aging, they experience dramatic shifts in their microenvironment. Bone marrow microenvironmental niches change for example in the production and levels of oncostatin M and osteopontin. As aging is a multi‐systemic process; the endocrine system is an especially good example. Expression of certain sex hormones, like estrogens E2 and E4 follow the life stages of human development. Sex differences in immunological responses both in disease and under homeostasis in humans [72], as well as post‐transplantation engraftment in mice [73], show that sexual dimorphism affects the hematopoietic system. Estrogen levels fluctuate throughout puberty, pregnancy, and aging, and both human and murine HSCs (alongside most blood cells types) express estrogen receptors ERα and ERβ, as well as G‐protein coupled estrogen receptor 1, suggesting that HSCs are sensitive to estrogen fluctuations [74]. Dose changes may affect tightly regulated cell fate decisions, which may lead to lineage skewing. Elevated estrogen levels during murine pregnancy promote cell division in HSCs, increased HSC frequency, cellularity, and erythropoiesis in the spleen [74], while premature menopause in women, associated with accelerated aging and defined by a drop in estrogen levels, has been linked to clonal hematopoiesis (CH) through DNMT3a [75]. Loss of estrogen also causes bone remodeling and reduction in endosteal niches, which leads to changes in the HSC microenvironment. Therefore, loss of HSC function during aging could be thought of as a response to the aging/changing niche. Taken together, these observations suggest that both HSCs and their bone marrow microenvironment are altered (co‐ordinately) through changing hormone levels, episodes of infection and inflammation, day/night light cycle changes, and most recently, nutritional deficiencies [76].

The second feature observed in the normal aging of the adult hematopoietic system is HSC fitness and competitive advantage leading to CH [67]. CH is defined by somatic mutation acquisition in HS/PCs. Whole exome sequencing reveal mutations in the DNA methylation genes DNMT3 and TET2 and the chromatin regulator ASXL1 that are associated with increasing age. These gene mutations comprised about 80% of the vast numbers of clones detected [77, 78] and are suggested to be drivers of CH in normal aging and are likely predisposition factors to leukemia [79]. Interestingly, deletion of Dnmt3a in mice, reveals an increase in HSC self‐renewal and B‐lymphoid bias, at the expense of differentiation [80]. Ongoing studies in DNMT3a W326R/+ mutant mice show the opposite HSC phenotype (G Neary, A. Jackson, personal communication). While aged‐related accumulation of such somatic mutations in HSCs appear to confer a selective growth advantage, the mechanisms by which this occurs is currently being investigated. One possible contributor is Oncostatin M signaling, which is elevated in Dnmt3a mutant aged HSCs and is thought to exhaust regulatory mechanisms that resolve inflammatory states in HSCs [81]. Another candidate interaction between HSCs and niche is suggested from studies comparing the potency of young and aged CD61 high/low expressing HSCs, that of osteopontin and CD61 [82]. CD61 is highly upregulated on aged HSCs while its ligand Osteopontin is downregulated in aged bone marrow. Osteopontin is known to be expressed by osteoblasts and osteoclasts. These findings suggest that such changes in the endosteal niche allow maintenance of only a subset of HSCs which could ultimately lead to competitive advantage and CH. Further somatic mutations caused by DNA damage (particularly in JAK2 and TP53 oncogenes) are thought to increase the propensity for the subsequent development of leukemia.

In conclusion, the healthy hematopoietic system is dynamic and changes throughout embryonic, fetal, neonatal, adolescent, reproductive and geriatric stages. It is represented by multiple layers of mature hematopoietic cells serving the organismal needs and diverse sets of progenitors and the fully potent self‐renewing cohort of HSCs that ensure a robust blood system during embryonic development, after birth and through adult life. By understanding the intrinsic and extrinsic changes occurring during aging, the rejuvenation of the blood system, although a difficult task, may at least in some part become a future reality.
